# Smaug/SAMD4A Restores Translational Activity of CUGBP1 and Suppresses CUG-Induced Myopathy

**DOI:** 10.1371/journal.pgen.1003445

**Published:** 2013-04-18

**Authors:** Maria de Haro, Ismael Al-Ramahi, Karlie R. Jones, Jerrah K. Holth, Lubov T. Timchenko, Juan Botas

**Affiliations:** 1 Department of Molecular and Human Genetics, Baylor College of Medicine, Houston, Texas, United States of America; 2 Jan and Dan Duncan Neurological Research Institute, Texas Children's Hospital, Houston, Texas, United States of America; 3 Department of Molecular Physiology and Biophysics, Baylor College of Medicine, Houston, Texas, United States of America; The Jackson Laboratory, United States of America

## Abstract

We report the identification and characterization of a previously unknown suppressor of myopathy caused by expansion of CUG repeats, the mutation that triggers Myotonic Dystrophy Type 1 (DM1). We screened a collection of genes encoding RNA–binding proteins as candidates to modify DM1 pathogenesis using a well established *Drosophila* model of the disease. The screen revealed *smaug* as a powerful modulator of CUG-induced toxicity. Increasing *smaug* levels prevents muscle wasting and restores muscle function, while reducing its function exacerbates CUG-induced phenotypes. Using human myoblasts, we show physical interactions between human Smaug (SMAUG1/SMAD4A) and CUGBP1. Increased levels of SMAUG1 correct the abnormally high nuclear accumulation of CUGBP1 in myoblasts from DM1 patients. In addition, augmenting SMAUG1 levels leads to a reduction of inactive CUGBP1-eIF2α translational complexes and to a correction of translation of MRG15, a downstream target of CUGBP1. Therefore, Smaug suppresses CUG-mediated muscle wasting at least in part via restoration of translational activity of CUGBP1.

## Author Summary

Myotonic dystrophy type 1 (DM1) is the most common among the muscular dystrophies causing muscle weakness and wasting in adults, and it is triggered by expansion of an untranslated CUG repeat. To identify potential therapeutic approaches, we used a *Drosophila* DM1 model to screen for genes capable of suppressing CUG-induced toxicity. Here we report that increased levels of the *smaug* gene prevent muscle wasting and, perhaps more impressively, also prevent muscle dysfunction caused by the DM1 mutation. Smaug interacts genetically and physically with CUGBP1, an RNA–binding protein previously implicated in DM1. We used myoblasts from DM1 patients and control individuals to investigate how Smaug suppresses CUG-induced myopathy. We found that increased human SMAUG1 (a.k.a. SMAD4A) levels revert the abnormal accumulation of CUGBP1 in myoblasts nuclei and restore normal translation of at least one mRNA regulated by CUGBP1 in the cytoplasm. These findings demonstrate that manipulating Smaug activity protects against the effects of the DM1 mutation, and they also support the idea that restoring normal CUGBP1 function is a potential therapeutic approach.

## Introduction

Myotonic Dystrophy type 1 (DM1) is a multisystemic neuromuscular disorder that has become a paradigm of a class of diseases caused by RNA toxicity. DM1 arises from expansion of a CTG triplet repeat in the 3′ untranslated region of the *DMPK* gene, and it accounts for the majority of adult cases of muscular dystrophy [1]–[5].

In DM1 the CUG-expanded mRNA is trapped in the nuclei where it forms nuclear foci and sequesters MBNL1 protein leading to loss of its activity [6], [7]. In addition, the mutant mRNA leads to increased steady-state levels of CUGBP1 (a.k.a CELF1) [8], [9] through its stabilization as a result of PKC phosphorylation [10]. Both MBNL1 and CUGBP1 are RNA-binding proteins involved in regulation of splicing [11]–[14], and aberrant splicing of the insulin receptor [12], muscle-specific chloride channel [13], [15] and many other genes [16], [17] occur in DM1.

The critical significance of MBNL1 sequestration for DM1 pathogenesis is eloquently demonstrated in loss of function and overexpression experiments. MBNL1 mutant mice show cataracts, myotonia, and other muscle abnormalities [7] that closely resemble a number of DM1 pathological features, and they also share many of the splicing aberrations observed in transgenic mice expressing CUG repeats [16], [17]. Importantly, MBNL1 overexpression ameliorates, muscle wasting in a *Drosophila* DM1 model [18], and myotonia and splicing aberrations in mouse models [19].

Evidence of the relevance of increased steady-state levels of CUGBP1 in DM1 pathogenesis comes from overexpression experiments. Transgenic mice expressing CUGBP1 show delays in muscle development and differentiation [20], muscle wasting [21], splicing misregulation [22] and DM1-like cardiac abnormalities [23]. Besides its nuclear role in splicing, CUGBP1 also has other functions in the cytoplasm including regulation of mRNA translation and stability [24]–[26]. Alterations of protein [25] and mRNA [16] levels occur in DM1 consistent with the idea that perturbation of CUGBP1 cytoplasmic functions contribute to DM1 pathogenesis. CUGBP1 cellular localization depends on its phosphorylation status [25]. Several kinases phosphorylate CUGBP1 at different residues and affect its localization within the cell. Activation of the Akt pathway increases CUGBP1 phosphorylation at Ser-28 altering the transition from proliferating myoblasts to differentiated myotubes in DM1 [27]. On the other hand, DM1 cells show decreased activity of cyclin D3-cdk4, another kinase that phosphorylates CUGBP1. This renders higher levels of unphosphorylated CUGBP1, which forms inactive complexes with eIF2α (CUGBP1-eIF2α) affecting translation of mRNAs required for myoblast differentiation. These inactive complexes containing CUGBP1 accumulate in the cytoplasm of DM1 cells in stress granules (SG) [25].

The richness of evidence implicating CUGBP1 in DM1 pathogenesis suggests the possibility that correcting the abnormal levels and activity of CUGBP1 may be a therapeutic approach to ameliorate DM1 pathogenesis. In support of this idea, Wang and colleagues used a pharmacological approach to inhibit PKC in mice expressing (CUG)_960_ in the heart; this treatment ameliorates the mortality rates and cardiac conduction as well as contractile abnormalities in this heart-specific DM1 mouse model [28]. Additional evidence comes from the observation that overexpression of a nuclear dominant negative CUGBP1 protein reverses dysregulation of a splicing minigene reporter in cultured cells, and of the CUGBP1 target *Nrap* exon 12 in DM1 mice [29].

Here we report that *smaug*, a gene not previously known to be implicated in DM1, is a powerful suppressor of CUG-induced myopathy when overexpressed in *Drosophila*. We show that human SMAUG1 protein (a.k.a SAMD4A) interacts with CUGBP1 and decreases its abnormally high steady-state levels in DM1 nuclei. Furthermore, increasing the levels of SMAUG1 in myoblasts of DM1 patients decreases the amount of inactive CUGBP1-eIF2α translational complexes. This suggests that SMAUG1 improves the activity of the CUGBP1-containing translational complexes that are dysfunctional in DM1, a hypothesis that is supported by data showing SMAUG1-mediated increased translation of the CUGBP1 translational target MRG15 in DM1 myoblasts.

## Results

### Increased levels of Smaug suppress CUG-induced myopathy and restore muscle function in *Drosophila*

To identify previously unknown genes implicated in DM1 pathogenesis, we used a well characterized *Drosophila* DM1 model [18]. Since DM1 is caused by expansion of an untranslated transcript, and MBNL1 and CUGBP1 are themselves RNA-binding proteins, we hypothesized that DM1 modifier genes may be enriched among genes encoding RNA binding proteins (RNA-BPs). Thus, we screened a collection of 93 loss of function and 17 overexpression alleles in 73 RNA-BP genes for their ability to modulate pathogenesis caused by expanded CUG repeats.

First, we used an external eye phenotype induced by expression of (CUG)_480_ as a primary screen to identify genes able to ameliorate or enhance CUG-induced toxicity. To validate the identified modifiers we tested the ability of the primary screen hits to modify CUG-induced muscle wasting.

**Figure 1:**
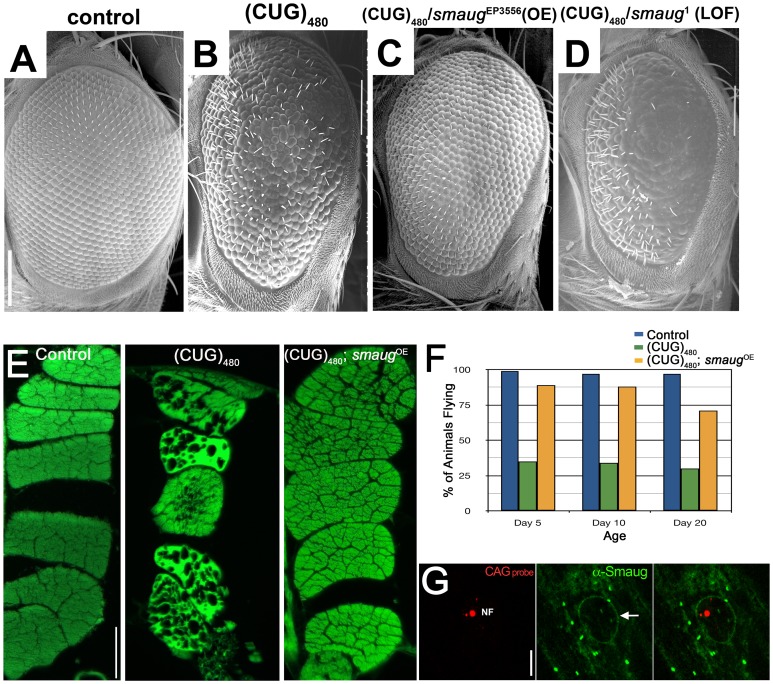
*smaug* overexpression suppresses expanded CUG-induced toxicity in *Drosophila*. A–D. Scanning electron microscopy (SEM) images of *Drosophila* eyes expressing (CUG)480 and different levels of *smaug*. A. Control GMR-Gal4 eye showing characteristic organization of ommatidia and interommatidial bristles. B. Animals expressing (CUG)480 under the control of GMR-Gal4 eye driver show reduced eye size, and disorganization of ommatidia and bristles. C. Overexpression of *smaug* ameliorates the (CUG)480-induced phenotypes. Note improved eye size and ommatidial organization relative to B. D. Reduced *smaug* function enhances (CUG)480-induced toxicity. Eyes of animals expressing (CUG)480 and carrying a heterozygous loss-of-function mutation in *smaug* show more severe eye size reduction, more ommatidial disorganization and less bristles than eyes of (CUG)480 animals (compare with B). E. Eosin stained transversal paraffin sections of indirect flight muscles (IFM). Expression of (CUG)480 in the muscles causes progressive loss of tissue and vacuolization. (CUG)480 animals of the same age that overexpress *smaug* show no muscle tissue loss or vacuolization. All E panels show IFMs from 20-day-old animals grown at 25C. F. Chart showing the percentage of control and experimental animals that are able to fly at different ages. Almost 100% of control animals (blue bars) are able to fly at days 5, 10 and 20. Animal expressing (CUG)480 in the IFMs show impaired flying ability (green bars). (CUG)480 animals that also overexpress *smaug* show rescue of the flying impairment phenotype (orange bars). n = 100. G. Larval muscles from animals expressing (CUG)480. probed with Cy3-labelled 5′-CAG-3′ LNA probe to detect nuclear foci (NF, in red) and stained with anti-Smaug (in green). Note that the Smaug protein does not localize to nuclear foci (NF). Smaug protein is mainly cytoplasmic where it accumulates in granules, and it also delineates the nucleus (arrow). Scale bar: A–D: 100 µm. E: 50 µm. G: 10 µm. Genotypes: A: *w*; GMR-Gal4/+. B: *w*; GMR-Gal4/+;*UAS-(CUG)480-M5T*/+. C: *w*; GMR-Gal4/+;*UAS-(CUG)480-M5T*/*smg*EP3556. D: *w*; GMR-Gal4/+;*UAS-(CUG)480-M5T*/*smg*1. E: Control: *w*; +; MHC-Gal4/+. (CUG)480: *w*; *UAS-(CUG)480-M5Q*, *UAS-(CUG)480-M13D*/+; MHC-Gal4/+. (CUG)480/*smgOE*: *w*; *UAS-(CUG)480-M5Q*, *UAS-(CUG)480-M13D*/+; MHC-Gal4/*smg*EP3556. F: Control: *w*; +; MHC-Gal4/+. (CUG)480: *w*; *UAS-(CUG)480-M13D*/+; MHC-Gal4/+. (CUG)480/smgOE: *w*; *UAS-(CUG)480-M13D*/+; MHC-Gal4/*smg*EP3556. G: *w*; *UAS-(CUG)480-M5Q*, *UAS-(CUG)480-M13D*/+; MHC-Gal4/+.

Among the RNA-BPs tested, we uncovered the *Drosophila* gene *smaug* as a strong modifier of both the eye and muscle degeneration. As shown in Figure 1 increased levels of Smaug rescue the eye disorganization and loss of bristle phenotypes induced by (CUG)_480_ expression (compare Figure 1C with 1B). Consistent with this result, partial loss of function of *smaug* caused by a heterozygous mutation enhances the (CUG)_480_-induced eye phenotype (compare Figure 1D with 1B). As shown in Figure S1 these overexpression and loss-of-function alleles do not induce any abnormal phenotypes in control animals that do not express expanded CUG repeats.

The DM1 *Drosophila* model shows progressive muscle wasting, which is easily studied in the indirect flight muscles of the thorax. While 1-day-old (CUG)_480_ flies have muscles that appear wild type, animals that are 20 days old show muscle disorganization, vacuolization and loss of muscle fibers [18]. We investigated the effect of increasing the levels of *smaug* on (CUG)_480_-induced muscle wasting. As shown in Figure 1E overexpression of *smaug* dramatically suppresses CUG-induced myopathy.

Next we investigated whether increased *smaug* levels could restore muscle function in addition to muscle integrity. Animals expressing (CUG)_480_ show a severe impairment in flying ability prior to showing any signs of muscle wasting by histological analysis (see green bars in Figure 1F). Increased levels of *smaug* sharply improve flying ability in animals expressing (CUG)_480_. (compare orange and green bars in Figure 1F). These muscle histology and behavioral data further support the idea that *smaug* is a suppressor of expanded-CUG toxicity in a variety of cellular contexts.

In addition, we investigated whether *Drosophila* Smaug protein and the expanded-CUG RNA co-localize in nuclear foci. Previous studies have shown that Smaug accumulates in cytoplasmic foci similar to stress granules, and that it can shuttle between the nucleus and the cytoplasm [30]. To determine whether Smaug protein localization is altered due to expression of (CUG)_480_, we performed in situ and immunofluorescense analysis of larval muscles of animals expressing (CUG)_480_. As shown in Figure 1G Smaug accumulates mainly in the cytoplasm in the form of granules (Figure 1G, green), and it does not co-localize with the nuclear CUG-containing foci (Figure 1G, red, NF). This observation suggests that the mechanism by which Smaug modulates expanded-CUG toxicity does not involve direct interaction with the nuclear foci.

### Smaug is a genetic interactor of CUGBP1

**Figure 2:**
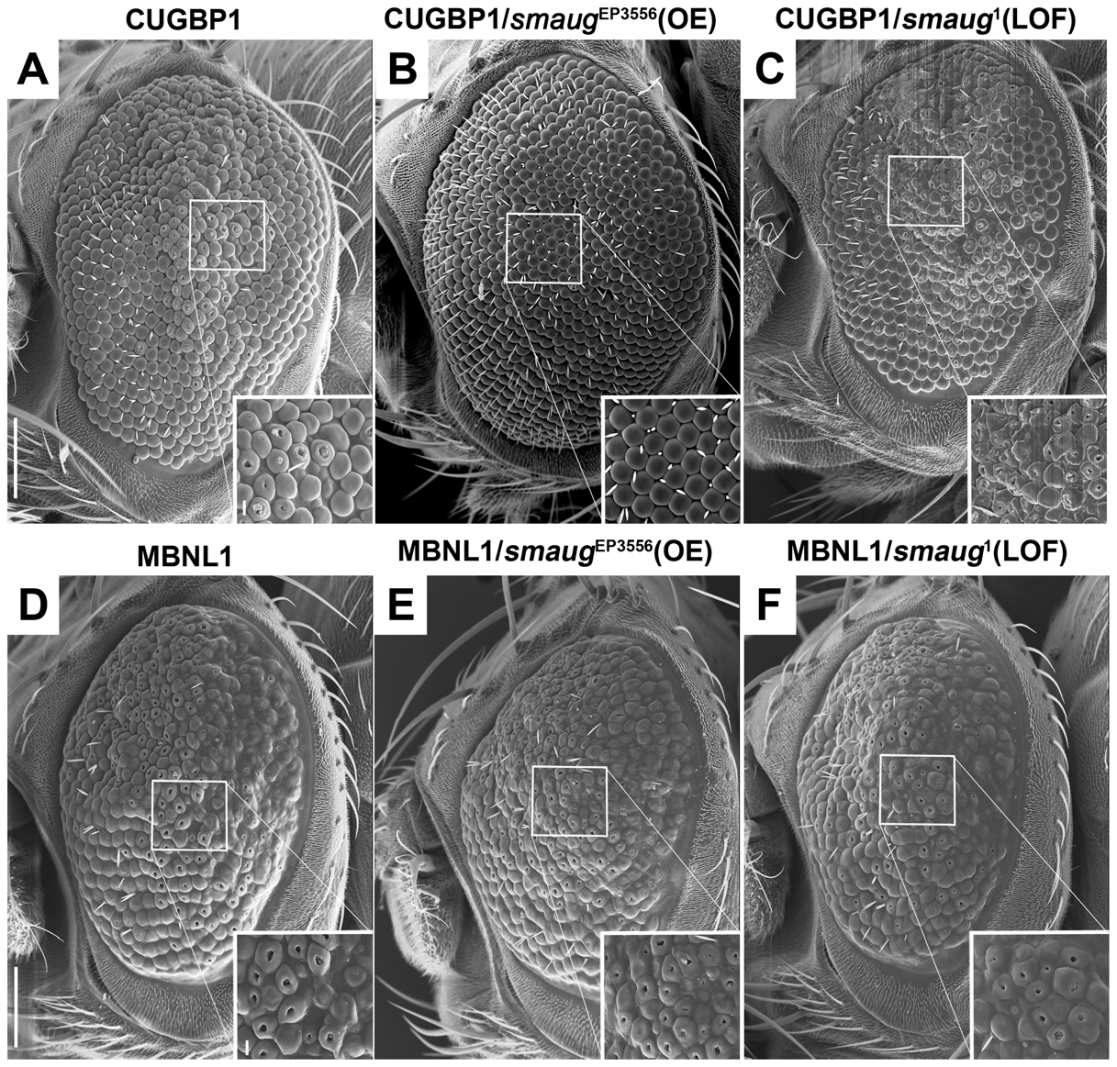
Smaug interacts genetically with CUGBP1 but not with MBNL1. A–C. SEM eye images of animals overexpressing CUGBP1 and different levels of *smaug*. A. SEM eye image showing that expression of CUGBP1 using GMR-Gal4 causes ommatidial disorganization, loss of bristles and decrease in eye size compared to control eyes (Figure 1A). This phenotype is most prominent in the central dorsal part of the eye (see close-up). B. *smaug* overexpression (OE) in animals expressing CUGBP1 rescues the ommatidial, bristle and eye size phenotypes. C. A heterozygous loss-of-function mutation in *smaug* enhances the CUGBP1-induced eye phenotype, particularly in the most affected central dorsal area of the eye (see close-up). D–F. SEM eye images of animals overexpressing MBNL1 and different levels of *smaug*. D. Expression of MBNL1 in the eye using GMR-Gal4 causes ommatidial disorganization, loss of bristles and reduction of eye size compared to control eyes (Figure 1A). Neither overexpression (E) nor partial loss of function (F) of *smaug* in animals expressing MBNL1 affect these phenotypes. Scale bar: A–F: 100 µm. Genotypes: A: *w*; GMR-Gal4/+; *UAS-CUGBP1-M2E*/+. B: *w*; GMR-Gal4/+; *UAS-CUGBP1-M2E*/*smg*EP3556. C: *w*; GMR-Gal4/+; *UAS-CUGBP1-M2E*/*smg*1. D: *w*; GMR-Gal4, *UAS-MBNL1-M6B*/+. E: w; GMR-Gal4, *UAS-MBNL1-M6B*/+; *smg*EP3556/+. F: *w*; GMR-Gal4, *UAS-MBNL1-M6B*/+; *smg*1/+.

The data described above and shown in Figure 1G does not suggest sequestration of Smaug in nuclear foci as a mechanism for Smaug modification of expanded-CUG toxicity. Consequently, we investigated possible interactions between *smaug* and the known key players in DM1 pathogenesis: MBNL1 and CUGBP1. Overexpression of human MBNL1 or CUGBP1 in the *Drosophila* eye leads to a mild disorganization phenotype [18], and Figure 2A and 2D. We used these phenotypes as assays to test potential genetic interactions with *smaug*. We found that *smaug* overexpression suppresses the phenotype induced by CUGBP1 expression (compare Figure 2B with Figure 2A). In addition, *smaug* partial loss of function enhances this phenotype (compare Figure 2C with Figure 2A). In contrast, altering *smaug* levels does not have an effect on the MBNL1-induced eye phenotype (Figure 2D–2F).

In summary, we find that CUGBP1 and Smaug interact genetically in *Drosophila*.

### *SMAUG1* overexpression reduces nuclear accumulation of CUGBP1, and both proteins physically interact and accumulate in cytoplasmic granules

**Figure 3:**
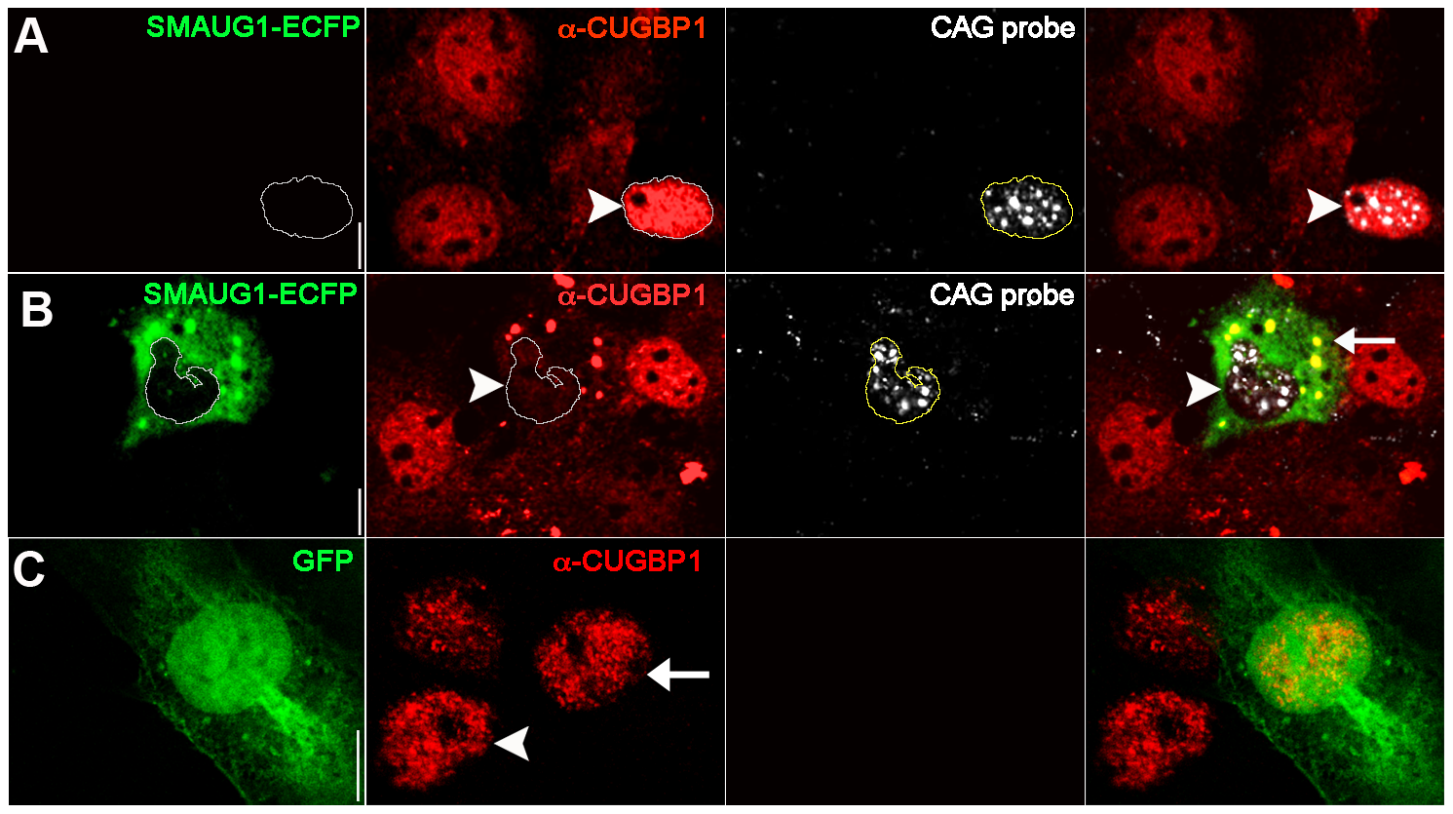
SMAUG1 expression reduces nuclear accumulation of CUGBP1, and both proteins co-localize in cytoplasmic granules. Immunofluorescense and in situ images of COSM6 cells. A. Expanded CUG repeat-transfected COSM6 cells show accumulation of the transcripts in nuclear foci (detected with a Cy3-labelled 5′-CAG-3′ LNA probe, white), and increased nuclear CUGBP1 signal (arrowhead) (α-CUGBP1, red). B. COSM6 cells co-transfected with CUGs and SMAUG1-ECFP show cytoplasmic signal of SMAUG1 (SMAUG1-ECFP, green) and CUG nuclear foci (CAG probe, white). These cells have decreased CUGBP1 nuclear signal (arrowhead) (α-CUGBP1, red); in addition, CUGBP1 co-localizes with SMAUG1 in cytoplasmic granules (arrow). C. Untransfected (arrowhead) and GFP-transfected (arrow) cells show similar nuclear CUGBP signal (α-CUGBP1, red). White lines in A–B delineate the nuclei. Scale bar: A–C: 10 µm.

To further investigate the interaction between SMAUG1 and CUGBP1, we performed immunofluorescense on COSM6 cells transfected with *SMAUG1* and (CUG)_960_. We found that CUGBP1 localizes predominantly in the nucleus in cells transfected only with (CUG)_960_ (see arrowhead in Figure 3A), an observation that is consistent with previous reports [8], [9], [31], [32]. We found, however, that nuclear CUGBP1 steady-state levels are significantly decreased in cells transfected with both (CUG)_960_ and *SMAUG1* (Figure 3B, arrowhead). CUGBP1 can be seen in these cells both diffuse in the cytoplasm as well as co-localizing with SMAUG1 in cytoplasmic granules (Figure 3B, arrow). As control we transfected with GFP and we could not observe differences in CUGBP1 signal between GFP-transfected (Figure 3C, arrow) and GFP-untransfected (Figure 3C, arrowhead) cells. A similar experiment was performed with MBNL1 and SMAUG1, but we found no evidence of changes in the accumulation of MBNL1 in nuclear foci following expression of *SMAUG1* (Figure S2).

**Figure 4:**
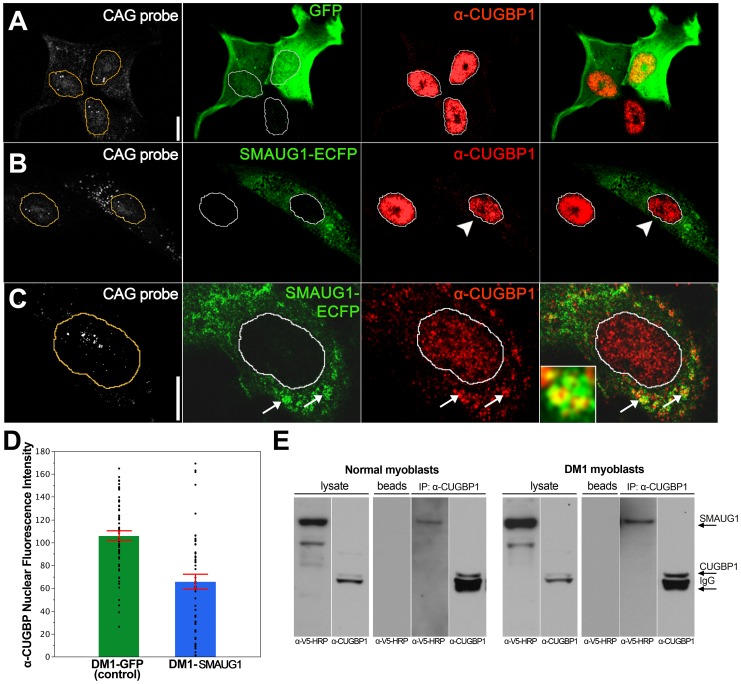
SMAUG1 and CUGBP1 co-localize and physically interact in DM1 myoblasts. A. Immunofluorescense and in situ images of DM1 myoblasts transfected with GFP. In control DM1 myoblasts transfected with GFP (green) CUGBP1 is predominantly in the nucleus (red); CUG foci are detected in the nuclei with a Cy3-labelled 5′-CAG-3′ LNA probe (CAG probe, white). B–C. Immunofluorescense and in situ images of DM1 myoblasts transfected with SMAUG1-ECFP. SMAUG1 is detected in the cytoplasm (SMAUG1-ECFP, green). Note that nuclear CUGBP1 signal (α-CUGBP1, red) is clearly diminished in DM1 myoblasts transfected with SMAUG1 (B, arrowhead). Longer exposure of CUGBP1 signal shows cytoplasmic CUGBP1 (C) and its co-localization with SMAUG1 in granules (arrows). D. Bar graph representing the intensity of CUGBP1 nuclear signal in DM1 myoblasts transfected with GFP (DM1-GFP, green bar), versus DM1 myoblasts transfected with SMAUG1 (DM1-SMAUG1, blue bar). Data was analyzed with ANOVA followed by Student's t test, p<0.0001. Black dots represent individual observations, red lines are the standard error of the mean. E. Western blot revealing co-immunoprecipitation between CUGBP1 and human SMAUG1 in extracts from SMAUG1-V5-transfected normal and DM1 human myoblasts. Pull down was carried out using anti-CUGBP1 antibody. SMAUG1 was visualized with anti-V5-HRP antibody. White lines in A–C delineate the nuclei. Scale bar: A–C: 10 µm.

To validate these data on a cell type more relevant to DM1, we investigated whether CUGBP1 distribution is altered by *SMAUG1* expression in myoblasts from DM1 patients. DM1 myoblasts transfected with GFP show predominantly nuclear CUGBP1 signal (Figure 4A). In contrast, DM1 myoblasts transfected with *SMAUG1* show significantly decreased levels of nuclear CUGBP1 (Figure 4B, compare intensity of CUGBP1 staining in SMAUG1-transfected (arrowhead) vs. untransfected myoblasts). We quantified the intensity of the signal of nuclear CUGBP1 staining in DM1 myoblasts transfected with SMAUG1 versus controls transfected with GFP, and we found that SMAUG1-transfected DM1 myoblasts show a significant decrease in the nuclear signal intensity compared to controls transfected with GFP (Figure 4D, p<0.0001).

**Figure 5:**
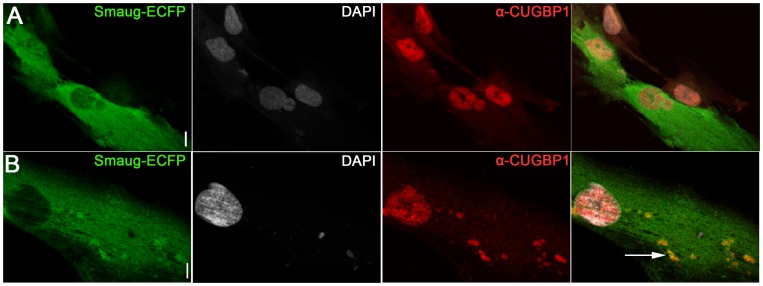
Expression of SMAUG1 in control human myoblasts does not affect CUGBP1 nuclear localization. A–B. Nuclear localization of CUGBP1 (α-CUGBP1, red) is not altered by expression of SMAUG1 (SMAUG1-ECFP, green) in control unaffected primary human myoblasts. CUGBP1 co-localizes with SMAUG1 cytoplasmic granules in control myoblasts (arrow in B). Scale bar: 10 µm.

**Figure 6:**
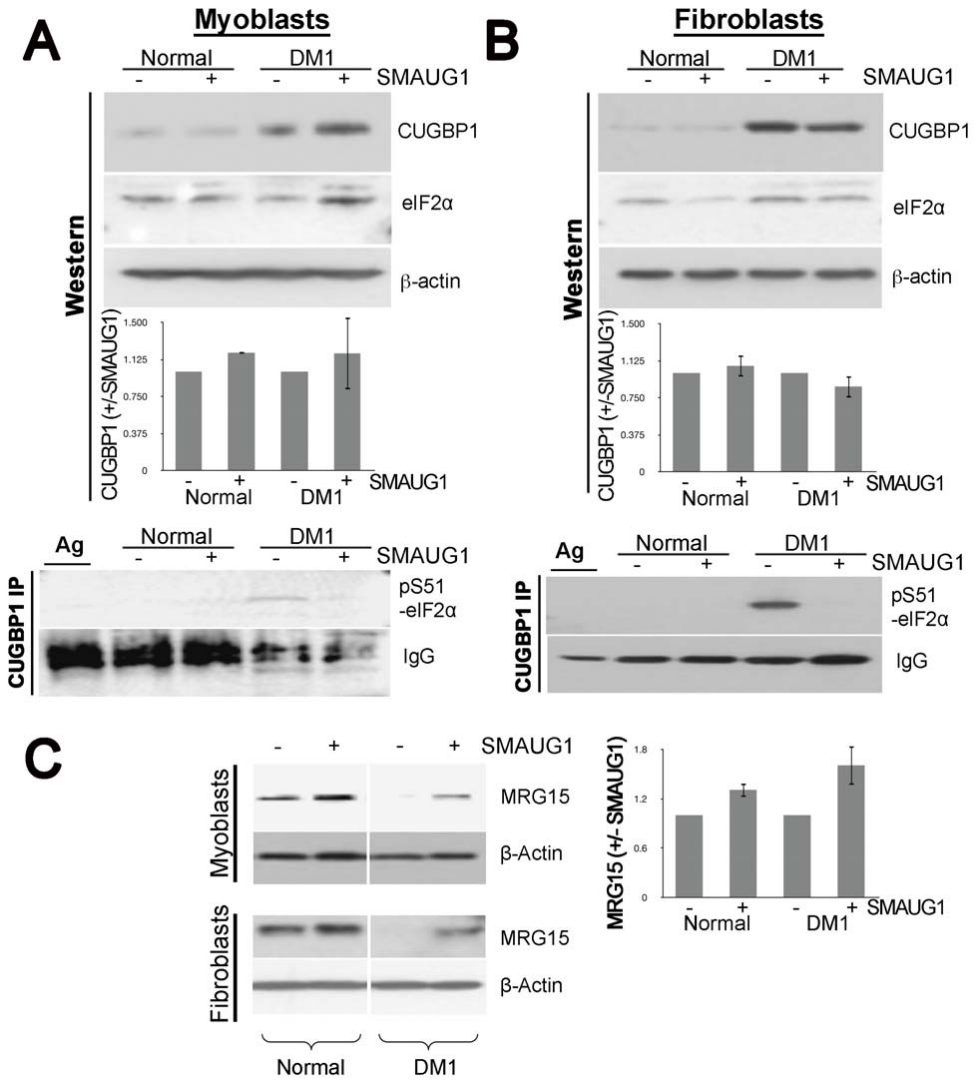
SMAUG1 reduces inactive CUGBP1/pS51-eIF2α translational complexes and recuperates normal levels of MRG15 protein in DM1 myoblasts and fibroblasts. A–B. Protein levels of total CUGBP1 and eIF2α in control and DM1 myoblasts (A) and fibroblasts (B) were detected by Western blotting of cytoplasmic fractions (Western). β-actin serves as a loading control. CUGBP1 levels are compared in untransfected and SMAUG1-transfected normal and DM1 myoblasts. Quantification is based in two experiments in myoblasts and two experiments in fibroblasts. Material immunoprecipitated with CUGBP1 antibodies was probed with antibody to specific inactive pS51-eIF2α (CUGBP1-IP). Note that inactive pS51-eIF2α is not detected after *SMAUG1* transfection. IgGs, heavy chains of immunoglobulins detected on the same filter. IP was repeated three times in myoblasts and three times in fibroblasts (see Figure S7). C. SMAUG1 recuperates normal levels of MRG15 protein in DM1 myoblasts and fibroblasts. Nuclear proteins of normal and DM1 myoblasts were examined by Western blotting with antibodies to MRG15. The filter was re-probed with antibodies to β-actin. The level of MRG15 in SMAUG1-transfected myoblasts are shown compared to untransfected normal and DM1 myoblasts from two experiments. Quantifications were performed using ImageJ Gel Analyzer software.

In addition we find that in SMAUG1-transfected DM1 myoblasts CUGBP1 and SMAUG1 co-localize in cytoplasmic granules (Figure 4C, arrows, and Figure S3). Cytoplasmic co-localization of both proteins was also observed in normal myoblast (Figure 5B). In spite of the observation that SMAUG1-expressing DM1 myoblasts show reduced nuclear CUGBP1, we did not detect an increase on cytoplasmic CUGBP1 in DM1 myoblasts transfected with SMAUG1 when compared to GFP-transfected controls (Figure S3, see also western blot of cytoplasmic fraction in Figure 6A). In control non-DM1 myoblasts transfected with SMAUG1 nuclear CUGBP1 signal remains the same (Figure 5).

Prompted by the genetic interaction between *Drosophila smaug* and human CUGBP1 and the co-localization of SMAUG1 and CUGBP1 in cells, we also investigated whether human SMAUG1/SAMD4A and CUGBP1 proteins physically interact. To do so, we performed co-immunoprecipitation experiments with cellular extracts from human myoblasts expressing SMAUG1. As shown in Figure 4E, SMAUG1 signal is detected after pull-down with anti-CUGBP1 antibody both in normal and DM1 myoblasts.

The intriguing finding that increased levels of SMAUG1 leads to decreased nuclear accumulation of CUGBP1 suggests that restoration of normal alternative splicing patterns may explain SMAUG1-mediated suppression of CUG-induced myopathy. To test this potential mechanism of SMAUG1 suppression, we examined the alternative splicing changes induced by either expanded CUG repeats or CUGBP1 overexpression.

Using a cTNT minigene reporter we found no evidence that overexpression of *SMAUG1* restores normal alternative splicing changes caused by either expanded CUG repeats or CUGBP1 overexpression (Figure S4).

### SMAUG1 reduces the amount of inactive CUGBP1-eIF2α translational complexes

Since we did not find evidence that increased SMAUG1 restore alternative splicing patterns, we investigated whether they restore CUGBP1 normal function in the cytoplasm. CUGPB1 regulates the translation and stability of mRNAs, and these activities are impaired in DM1 [24]–[26]; thus, we asked if the translational activity of CUGPB1 is influenced by SMAUG1.

In the cytoplasm, CUGBP1 interacts with eukaryotic translation initiation factor eIF2α (eIF2α), and its translational activity is mediated by CUGBP1-eIF2α complexes [33]. CUGBP1 phosphorylated at S302 binds to unphosphorylated eIF2α (non-pS51-eIF2α) making active CUGBP1-eIF2α translational complexes, whereas CUGBP1 unphosphorylated at S302 binds with higher affinity to inactive pS51-eIF2α forming CUGBP1-eIF2α inactive translational complexes [25]. In DM1 cells, the levels of inactive eIF2α (pS51-eIF2α) are increased, and formation of inactive CUGBP1-eIF2α complexes inhibits translation of certain mRNAs in DM1 myoblasts [25].

Therefore, we examined the effects of SMAUG1 on the abundance of inactive CUGBP1-eIF2α complexes. Western blot analysis of cytoplasmic extracts from transfected normal and DM1 myoblasts and fibroblasts show that the total cytoplasmic levels of CUGBP1 are increased in DM1 myoblasts and fibroblasts, and are not affected significantly by SMAUG1 (Figure 6A–6B and Figure S5). Additionally, we investigated if levels of total eIF2α are altered by SMAUG1 expression in cytoplasm. As shown in Figure 6A and 6B, total levels of eIF2α remain unchanged upon SMAUG1 transfection in both normal and DM1 myoblasts and fibroblasts. We then performed co-IP experiments on cytoplasmic protein extracts from normal and DM1 myoblasts and fibroblasts to test whether SMAUG1 expression alters the levels of inactive CUGBP1-eIF2α complexes. We found that in control GFP-transfected DM1 myoblasts/fibroblasts pS51-eIF2α-CUGBP1 inactive complexes are abundant. This is in striking contrast to SMAUG1-transfected DM1 myoblasts (Figure 6A, see CUGBP1-IP) and Figure S6) and fibroblasts (Figure 6B, see CUGBP1-IP) and Figure S6) where these complexes are undetectable. Thus, increasing SMAUG1 levels decreases the steady-state levels of CUGBP1-eIF2α inactive translational complexes.

### SMAUG1 restores translation of CUGBP1 target MRG15 in DM1 myoblasts

Previous reports have shown that MRG15 mRNA translation is controlled by CUGBP1-eIF2α complexes. Particularly, inactive CUGBP1-eIF2α complexes trap MRG15 mRNA in stress granules and reduces protein levels of MRG15 in DM1 compared to normal myoblasts [25]. Since we found that expression of SMAUG1 reduces amounts of inactive CUGBP-eIF2α complexes, we investigated if this reduction corrects translation of MRG15, a target of the CUGBP-eIF2α complex. Western blot analysis of nuclear protein extracts shows that DM1 cells contain reduced amounts of MRG15; however, SMAUG1 restores translation of MRG15 in DM1 cells to near normal levels in both myoblasts and fibroblasts (Figure 6C, Figure S7). In summary, these data indicate that expression of SMAUG1 significantly reduces the amounts of inactive CUGBP1-eIF2α complexes and enhances translation of MRG15.

## Discussion

Here we show that increased expression levels of *smaug*, a conserved gene involved in translational regulation, suppresses CUG-induced muscle wasting and, notably, it also restores normal muscle function in a *Drosophila* model of DM1. Experiments in DM1 myoblasts indicate that the human homolog *SMAUG1/SAMD4A* suppresses the toxic effects of expanded CUG repeats at least in part by restoring impaired CUGBP1 translational functions.

Early during DM1 pathogenesis CUGBP1 steady-state levels increase as a consequence of PKC-mediated phosphorylation [10]. This leads to disrupted regulation of alternative splicing, as well as impairments in mRNA stability and mRNA translation, all of which contribute to the multiple features of the disease (reviewed in [34], [35]). In addition, CUGBP1 overexpression in wild-type mice mimics some of the functional, histopathological and molecular features of DM1 [22], [36], [37], while CUGBP1 overexpression in *Drosophila* enhances expanded-CUG induced pathology [18]. Together these observations suggest that restoring normal CUGBP1 levels and activities may reverse DM1 pathology. This approach however may prove difficult to execute. First, there are several CUGBP1-like proteins in mammals and in *Drosophila* making proof-of-principle experiments using loss-of-function alleles complicated. To circumvent the problem of functional redundancy, a dominant-negative CUGBP1 construct was expressed in culture cells and mice, and this resulted in the reversion of abnormal alternative splicing [29]. Expression of dominant-negative CUGBP1, however, also leads to cardiac and skeletal muscle pathology [38], [39]. A second and perhaps more important caveat is that CUG expansion leads to increased nuclear levels of CUGBP1 [8], [40] (i.e., a gain of function), while in the cytoplasm the same mutation leads to the inactivation of CUGBP1 translational complexes [25] (i.e., loss of function). Hence, restoring normal CUGBP1 activities in both nucleus and cytoplasm by modulating CUGBP1 itself seems challenging. An alternative approach is to target other factors modulating CUGBP1 function. One such factor is PKC, a kinase that phosphorylates and stabilizes CUGBP1 [10]. Indeed, PKC pharmacological inhibition ameliorates the cardiac phenotypes in a heart-specific DM1 mouse model [28].

The data presented here reveals that *SMAUG1/SAMD4* is able to restore CUGBP1 normal levels and activities. We found that increasing the levels of SMAUG1 leads to decreased levels of nuclear CUGBP1 in DM1 myoblasts. This intriguing observation suggested that rescue of alternative splicing alterations may be a possible mechanism to explain the observed suppression of CUG-induced myopathy. This is an open possibility because even though we did not find evidence of SMAUG1 modulating splicing on a cTNT minigene, we cannot rule out its effects on other unknown splicing targets.

We showed that SMAUG1 can re-activate impaired CUGBP1 translational activities in the cytoplasm. *smaug* was first discovered in *Drosophila* as a translational regulator of *nanos* mRNA in the posterior pole of the embryo [41]. In this context it functions as a translational repressor by capturing transcripts containing Smaug recognition elements, forming stable ribonucleoprotein particles, and displacing the eIF4G initiation factor [42]. Smaug also promotes the destabilization and degradation of *nanos* mRNA by recruiting a deadenylation factor [43]–[45]. There are two *smaug* homologous genes in mammals [30]. One of them, *SMAUG1/SAMD4A*, forms mRNA-silencing foci at postsynapses of hippocampal neurons that respond to NMDA and modulate synapse formation [46]. We find that SMAUG1 has a positive function in the context of CUGBP1-dependent translation in myoblasts suggesting that SMAUG1 is not a dedicated repressor of translation, but rather a translational regulator whose function is context dependent.

In DM1, high levels of CUGBP1 unphosphorylated at S302 form inactive translational complexes with pS51-eIF2α. We found that increased levels of SMAUG1 lead to a dramatic reduction of CUGBP1-eIF2α inactive complexes. It is unlikely that this is a result of nuclear CUGBP1 being exported to the cytoplasm because we did not detect an increase of CUGBP1 in western blots of cytoplasmic extracts from SMAUG1-transfected DM1 myoblasts or fibroblasts. This observation was confirmed by immnofluorescence experiments showing similar levels of cytoplasmic CUGBP1 between SMAUG1-transfected and GFP-transfected DM1 myoblasts. An attractive possibility is that the interaction between SMAUG1 and CUGBP1 promotes repair of defective initiation complexes. In support of this hypothesis we observe an increase in translation of MRG15. Translation of MEF2A, C/EBPbeta, p21, and other translational CUGBP1 targets such as cyclin D1 and HDAC1 are promoted by active CUGBP1/elF2 complexes (i.e., formed by p-S302-CUGBP1 and elF2 not phosphorylated at S51). However, we only know of one target, MRG15, whose translation is inhibited by inactive CUGBP1/elF2 complexes (i.e., formed by CUGBP1 not phosphorylated at S302, and p-S51-elF2) [25]. Thus, we expect that other mRNA targets of CUGBP1 whose translation is impaired in DM1 may be corrected as well; however, these other mRNAs have not been identified yet.

The only therapy available for DM1 patients is used to treat the symptoms rather than the cause of the disease. Efforts to develop therapeutic avenues for DM1 pathogenesis include: 1) to revert the instability of the expansion, 2) to target the toxic RNA with ribozymes or antisense oligonucleotides [47]–[51], 3) to target the CUG RNA hairpins with siRNA [52]. Potential alternatives are to develop therapeutic approaches to restore CUGBP1 and MBNL1 protein levels and activities [18], [19], [28] (reviewed in [53]). The data reported here suggests that therapeutics designed to increase SMAUG1 protein levels could be useful to ameliorate the toxicity of the mutant RNA in DM1.

## Materials and Methods

### Fly strains

The transgenic lines UAS-(CTG)_480_, UAS-*MBNL1*, and UAS-*CUGBP1* have been previously described [18]. *Mhc*-GAL4 was obtained from G. Davis (UCSF). *gmr*-GAL4, *smg* ^1^ and *smg* ^EP3556^ were obtained from Bloomington Stock Center (Indiana) and Szeged Stock Center (Hungary).

### Scanning electron microscopy of *Drosophila* eyes and muscle paraffin sections of *Drosophila* adult thoraxes

Processing of flies for SEM and image acquisition were performed following previously published procedures [54].

For paraffin sections, adult thoraxes were dissected out, fixed overnight in 4% formaldehyde in PBS, washed in PBS and dehydrated in increasing concentrations of ethanol. Thoraxes were embedded in paraffin. Serial sections of 10 µm were obtained and rehydrated to water. Sections were stained with eosin (Sigma) and the fluorescent images were captured using an AxioCam MRc camera (Zeiss) attached to a Microphot-FXA microscope (Nikon).

### Flying assay

Individual adult flies were dropped one at a time from the top of a 12-inch cylinder and the landing position in the cylinder was recorded. One hundred flies per genotype were scored and each fly was tested three times.

### *In situ* hybridization and immunofluorescense of *Drosophila* larval muscles

The protocols were previously described in [18]. Anti-Smaug antibody (provided by C.A. Smibert) was used at a concentration of 1∶50.

### Cell culture *in situ* and immunofluorescense analysis

Constructs used for transfection were (CUG)_960_ (T.A. Cooper) and SMAUG1-ECFP (G.L. Boccaccio). COSM6 cells were transfected with (CUG)_960_ alone or together with SMAUG1-ECFP using Amaxa Cell Line Nucleofector Line R (Lonza). Two days after transfection cells were fixed in 4% formaldehyde for one hour, washed and hybridized with a Cy3-labelled 5′-CAG-3′ LNA probe for one hour, followed by incubation with mouse anti-CUGBP1 3B1 antibody (1∶120) overnight at 4C. Secondary anti-mouse antibody labelled with Cy5 was used to visualize CUGBP1.

Human primary myoblasts derived from control individuals or from DM1 patients with 300 CTG repeats were grown for no more than 12 passages and transfected with SMAUG1-ECFP or control pmaxGFP using Amaxa Cell Line Nucleofector Line NHDF (Lonza). Two days after transfection in situ and immunofluorescense was performed as described in the above paragraph.

For quantification of CUGBP1 nuclear signal, pictures taken at the confocal microscope under the same conditions were analyzed using ImageJ software. Pictures of at least 50 different cells were taken. Data sets were compared using ANOVA followed by Student's t analysis.

### Western blot and co-immunoprecipitation of human myoblasts and fibroblasts

Transfection of myoblasts and fibroblasts with SMAUG1-V5 (G.L. Boccaccio) was performed using Amaxa Nucleofector Line NHDF (Lonza). For co-immunoprecipitation of myoblasts in Figure 4E, anti-CUGBP1 3B1 antibody (Novus Biologicals) was coupled to Dynabeads M-270 Epoxy (Invitrogen), and co-IP was performed with Dynabeads Co-Immunoprecipitation kit (Invitrogen) using anti-V5 (Invitrogen) antibodies.

For western blot analysis, control and DM1 myoblasts (300 CTG repeats) and fibroblasts (2000 and 1600 CTGs) were transfected as described above. Two days after transfection nuclear and cytoplasmic protein fractions were extracted [31]. Twenty five µg of cytoplasmic proteins were separated by gel electrophoresis, transferred on membrane and incubated with anti-CUGBP 3B1 and anti-eIF2α (FL-315, Santa Cruz, CA, USA).

Co-IP with 100 µg of cytoplasmic protein was performed using the protocol associated with Trueblot Antibodies from eBioscience. Antibody for pS51-eIF2α was S51-sc12412-R from Santa Cruz.

For detection of MRG15, nuclear protein fractions of cells transfected with SMAUG1 or GFP were separated by gel electrophoresis, transferred on membrane and incubated with anti-MRG15 (F-19) and anti-β-actin (AC-15) from Santa Cruz.

## Supporting Information

Figure S1 Overexpression and loss-of-function alleles of *smaug* do not modify the phenotype of control eyes. A–B. Neither overexpression (A), nor loss-of-function (B) alleles of *smaug* in heterozygosity cause an abnormal phenotype in control eyes (GMR-Gal4).
Genotypes: A: *w*; GMR-Gal4/+; *smg*EP3556/+. B: *w*; GMR-Gal4/+; *smg*1/+. Scale bar: 100 µm.
(TIF)

Figure S2 MBNL1 sequestration in nuclear foci is not altered by SMAUG1 overexpression. Immunofluorescense and in situ images of COSM6 cells A. In cells transfected with (CUG)960, the expanded CUGs accumulate in the nuclear foci (CAG probe, white), where they sequester endogenous MBNL1 (α-MBNL1, red). B. In cells transfected with SMAUG1-ECFP, SMAUG1-ECFP (green) accumulates in the cytoplasm, where it co-localizes with MBNL1 (arrowheads) (α-MBNL1, red). C. In cells cells co-transfected with (CUG)960 and SMAUG1-ECFP, sequestration of MBNL1 (α-MBNL1, red) by CUG nuclear foci (CAG probe, white) is shown by co-localization of both (arrow). Note that co-localization of MBNL1 and CUG nuclear foci is not altered by SMAUG1-ECFP (green) expression (compare panels A and C). Scale bar: 10 µm.
(TIF)

Figure S3 Expression of SMAUG1 in DM1 human myoblasts does not affect cytoplasmic CUGBP1 levels. A. Bar graph representing the intensity of α-CUGBP1 cytoplasmic signal in DM1 myoblasts transfected with GFP (DM1-GFP, green bar), versus DM1 myoblasts transfected with SMAUG1 (DM1-SMAUG1, blue bar). Data was analyzed with ANOVA followed by Student's t test; NS: not significant. B–C. Representative images of DM1 myoblasts transfected with GFP (green) (B) or SMAUG1 (SMAUG1-ECFP, green) (C) taken at high exposure to reveal cytoplasmic CUGBP1. Note that the intensity of cytoplasmic CUGBP1 (α-CUGBP1, red) is similar in both cases. See also co-localization of CUGBP1 and SMAUG1 (C, arrow). Scale bar: 10 µm.
(TIF)

Figure S4 SMAUG1 does not modify splicing of cTNT minigene. A. SMAUG1 does not modify splicing changes caused by CUGBP1 or (CTG)960 on cTNT minigene. CosM6 cells transfected with cTNT minigene show 66% exon 5 inclusion. Upon co-transfection with CUGBP1 or (CTG) 960 exon 5 inclusion increases to 92 and 82% respectively. This splicing pattern is not affected by SMAUG1 transfection. Expression of SMAUG1 alone with the minigene has a similar splicing pattern to cTNT minigene alone with 61% exon inclusion.
(TIF)

Figure S5 SMAUG1 does not affect abnormally elevated levels of CUGBP1 in the cytoplasm. A. Western blot analysis of protein levels from cytoplasmic extracts in control and DM1 myoblasts. CUGBP1 levels are increased in DM1 myoblasts. B. Western blot analysis of protein levels from cytoplasmic extracts in control and DM1 fibroblasts. CUGBP1 levels are increased in DM1 fibroblasts. β-Actin used as control.
(TIF)

Figure S6 Reproduction of the observation that SMAUG1 reduces inactive CUGBP1/pS51-eIF2α translational complexes. A. Cytoplasmic protein extracts from normal and DM1 myoblasts immunoprecipitated with CUGBP1 antibody was probed with antibody to specific inactive pS51-eIF2α (CUGBP1 IP) in two additional experiments with similar results. B. Cytoplasmic protein extracts from normal and DM1 fibroblasts immunoprecipitated with CUGBP1 antibody was probed with antibody to specific inactive pS51-eIF2α (CUGBP1 IP) in two additional experiments with similar results. Note that inactive pS51-eIF2α is undetectable after SMAUG1 transfection. IgG, heavy chains of immunoglobulins detected on the same filter.
(TIF)

Figure S7 Reproduction of the observation that SMAUG1 recuperates normal levels of MRG15 protein in DM1 myoblasts and fibroblasts. SMAUG1 recuperates normal levels of MRG15 protein in DM1 myoblasts and fibroblasts. Nuclear and cytoplasmic proteins of normal and DM1 myoblasts and fibroblasts were examined by Western blotting with antibodies to MRG15. The filter was re-probed with antibodies to β-actin.
(TIF)

## References

[1] Harper PS, Brook JD, Newman EE (2001) Myotonic dystrophy. London: W. B. Saunders. ix, 436 p. p.

[2] Osborne RJ, Thornton CA (2006) RNA-dominant diseases. Hum Mol Genet 15 Spec No 2: R162–169. 16987879 10.1093/hmg/ddl181

[3] Ranum LP, Cooper TA (2006) RNA-mediated neuromuscular disorders. Annu Rev Neurosci 29: 259–277. 16776586 10.1146/annurev.neuro.29.051605.113014

[4] Schoser B, Timchenko L (2010) Myotonic dystrophies 1 and 2: complex diseases with complex mechanisms. Curr Genomics 11: 77–90. 20885816 10.2174/138920210790886844 PMC2874224

[5] Sicot G, Gourdon G, Gomes-Pereira M (2011) Myotonic dystrophy, when simple repeats reveal complex pathogenic entities: new findings and future challenges. Hum Mol Genet 20: R116–123. 21821673 10.1093/hmg/ddr343

[6] Miller JW, Urbinati CR, Teng-Umnuay P, Stenberg MG, Byrne BJ, et al (2000) Recruitment of human muscleblind proteins to (CUG)(n) expansions associated with myotonic dystrophy. EMBO J 19: 4439–4448. 10970838 10.1093/emboj/19.17.4439 PMC302046

[7] Kanadia RN, Johnstone KA, Mankodi A, Lungu C, Thornton CA, et al (2003) A muscleblind knockout model for myotonic dystrophy. Science 302: 1978–1980. 14671308 10.1126/science.1088583

[8] Wang GS, Kearney DL, De Biasi M, Taffet G, Cooper TA (2007) Elevation of RNA-binding protein CUGBP1 is an early event in an inducible heart-specific mouse model of myotonic dystrophy. J Clin Invest 117: 2802–2811. 17823658 10.1172/JCI32308 PMC1964514

[9] Timchenko NA, Cai ZJ, Welm AL, Reddy S, Ashizawa T, et al (2001) RNA CUG repeats sequester CUGBP1 and alter protein levels and activity of CUGBP1. J Biol Chem 276: 7820–7826. 11124939 10.1074/jbc.M005960200

[10] Kuyumcu-Martinez NM, Wang GS, Cooper TA (2007) Increased steady-state levels of CUGBP1 in myotonic dystrophy 1 are due to PKC-mediated hyperphosphorylation. Mol Cell 28: 68–78. 17936705 10.1016/j.molcel.2007.07.027 PMC2083558

[11] Philips AV, Timchenko LT, Cooper TA (1998) Disruption of splicing regulated by a CUG-binding protein in myotonic dystrophy. Science 280: 737–741. 9563950 10.1126/science.280.5364.737

[12] Savkur RS, Philips AV, Cooper TA (2001) Aberrant regulation of insulin receptor alternative splicing is associated with insulin resistance in myotonic dystrophy. Nat Genet 29: 40–47. 11528389 10.1038/ng704

[13] Charlet BN, Savkur RS, Singh G, Philips AV, Grice EA, et al (2002) Loss of the muscle-specific chloride channel in type 1 myotonic dystrophy due to misregulated alternative splicing. Mol Cell 10: 45–53. 12150906 10.1016/s1097-2765(02)00572-5

[14] Ho TH, Charlet BN, Poulos MG, Singh G, Swanson MS, et al (2004) Muscleblind proteins regulate alternative splicing. EMBO J 23: 3103–3112. 15257297 10.1038/sj.emboj.7600300 PMC514918

[15] Lueck JD, Mankodi A, Swanson MS, Thornton CA, Dirksen RT (2007) Muscle chloride channel dysfunction in two mouse models of myotonic dystrophy. J Gen Physiol 129: 79–94. 17158949 10.1085/jgp.200609635 PMC2151606

[16] Osborne RJ, Lin X, Welle S, Sobczak K, O'Rourke JR, et al (2009) Transcriptional and post-transcriptional impact of toxic RNA in myotonic dystrophy. Hum Mol Genet 18: 1471–1481. 19223393 10.1093/hmg/ddp058 PMC2664149

[17] Du H, Cline MS, Osborne RJ, Tuttle DL, Clark TA, et al (2010) Aberrant alternative splicing and extracellular matrix gene expression in mouse models of myotonic dystrophy. Nat Struct Mol Biol 17: 187–193. 20098426 10.1038/nsmb.1720 PMC2852634

[18] de Haro M, Al-Ramahi I, De Gouyon B, Ukani L, Rosa A, et al (2006) MBNL1 and CUGBP1 modify expanded CUG-induced toxicity in a Drosophila model of myotonic dystrophy type 1. Hum Mol Genet 15: 2138–2145. 16723374 10.1093/hmg/ddl137

[19] Kanadia RN, Shin J, Yuan Y, Beattie SG, Wheeler TM, et al (2006) Reversal of RNA missplicing and myotonia after muscleblind overexpression in a mouse poly(CUG) model for myotonic dystrophy. Proc Natl Acad Sci U S A 103: 11748–11753. 16864772 10.1073/pnas.0604970103 PMC1544241

[20] Timchenko NA, Patel R, Iakova P, Cai ZJ, Quan L, et al (2004) Overexpression of CUG triplet repeat-binding protein, CUGBP1, in mice inhibits myogenesis. J Biol Chem 279: 13129–13139. 14722059 10.1074/jbc.M312923200

[21] Ward AJ, Rimer M, Killian JM, Dowling JJ, Cooper TA (2010) CUGBP1 overexpression in mouse skeletal muscle reproduces features of myotonic dystrophy type 1. Hum Mol Genet 19: 3614–3622. 20603324 10.1093/hmg/ddq277 PMC2928132

[22] Ho TH, Bundman D, Armstrong DL, Cooper TA (2005) Transgenic mice expressing CUG-BP1 reproduce splicing mis-regulation observed in myotonic dystrophy. Hum Mol Genet 14: 1539–1547. 15843400 10.1093/hmg/ddi162

[23] Koshelev M, Sarma S, Price RE, Wehrens XH, Cooper TA (2010) Heart-specific overexpression of CUGBP1 reproduces functional and molecular abnormalities of myotonic dystrophy type 1. Hum Mol Genet 19: 1066–1075. 20051426 10.1093/hmg/ddp570 PMC2830830

[24] Timchenko NA, Wang GL, Timchenko LT (2005) RNA CUG-binding protein 1 increases translation of 20-kDa isoform of CCAAT/enhancer-binding protein beta by interacting with the alpha and beta subunits of eukaryotic initiation translation factor 2. J Biol Chem 280: 20549–20557. 15788409 10.1074/jbc.M409563200

[25] Huichalaf C, Sakai K, Jin B, Jones K, Wang GL, et al (2010) Expansion of CUG RNA repeats causes stress and inhibition of translation in myotonic dystrophy 1 (DM1) cells. FASEB J 24: 3706–3719. 20479119 10.1096/fj.09-151159 PMC2996918

[26] Lee JE, Lee JY, Wilusz J, Tian B, Wilusz CJ (2010) Systematic analysis of cis-elements in unstable mRNAs demonstrates that CUGBP1 is a key regulator of mRNA decay in muscle cells. PLoS ONE 5: e11201 doi:10.1371/journal.pone.0011201. 20574513 10.1371/journal.pone.0011201 PMC2888570

[27] Salisbury E, Sakai K, Schoser B, Huichalaf C, Schneider-Gold C, et al (2008) Ectopic expression of cyclin D3 corrects differentiation of DM1 myoblasts through activation of RNA CUG-binding protein, CUGBP1. Exp Cell Res 314: 2266–2278. 18570922 10.1016/j.yexcr.2008.04.018 PMC2494712

[28] Wang GS, Kuyumcu-Martinez MN, Sarma S, Mathur N, Wehrens XH, et al (2009) PKC inhibition ameliorates the cardiac phenotype in a mouse model of myotonic dystrophy type 1. J Clin Invest 119: 3797–3806. 19907076 10.1172/JCI37976 PMC2786786

[29] Berger DS, Ladd AN (2011) Repression of nuclear CELF activity can rescue CELF-regulated alternative splicing defects in skeletal muscle models of myotonic dystrophy. PLoS Curr 4: RRN1305 doi:10.1371/currents.RRN1305. 22453899 10.1371/currents.RRN1305 PMC3286860

[30] Baez MV, Boccaccio GL (2005) Mammalian Smaug is a translational repressor that forms cytoplasmic foci similar to stress granules. J Biol Chem 280: 43131–43140. 16221671 10.1074/jbc.M508374200

[31] Timchenko LT, Miller JW, Timchenko NA, DeVore DR, Datar KV, et al (1996) Identification of a (CUG)n triplet repeat RNA-binding protein and its expression in myotonic dystrophy. Nucleic Acids Res 24: 4407–4414. 8948631 10.1093/nar/24.22.4407 PMC146274

[32] Roberts R, Timchenko NA, Miller JW, Reddy S, Caskey CT, et al (1997) Altered phosphorylation and intracellular distribution of a (CUG)n triplet repeat RNA-binding protein in patients with myotonic dystrophy and in myotonin protein kinase knockout mice. Proc Natl Acad Sci U S A 94: 13221–13226. 9371827 10.1073/pnas.94.24.13221 PMC24290

[33] Timchenko LT, Salisbury E, Wang GL, Nguyen H, Albrecht JH, et al (2006) Age-specific CUGBP1-eIF2 complex increases translation of CCAAT/enhancer-binding protein beta in old liver. J Biol Chem 281: 32806–32819. 16931514 10.1074/jbc.M605701200

[34] Wheeler TM, Thornton CA (2007) Myotonic dystrophy: RNA-mediated muscle disease. Curr Opin Neurol 20: 572–576. 17885447 10.1097/WCO.0b013e3282ef6064

[35] Lee JE, Cooper TA (2009) Pathogenic mechanisms of myotonic dystrophy. Biochem Soc Trans 37: 1281–1286. 19909263 10.1042/BST0371281 PMC3873089

[36] Koshelev M, Sarma S, Price RE, Wehrens XH, Cooper TA (2010) Heart-specific overexpression of CUGBP1 reproduces functional and molecular abnormalities of myotonic dystrophy type 1. Hum Mol Genet 19: 1066–1075. 20051426 10.1093/hmg/ddp570 PMC2830830

[37] Ward AJ, Rimer M, Killian JM, Dowling JJ, Cooper TA (2010) CUGBP1 overexpression in mouse skeletal muscle reproduces features of myotonic dystrophy type 1. Hum Mol Genet 19: 3614–3622. 20603324 10.1093/hmg/ddq277 PMC2928132

[38] Ladd AN, Taffet G, Hartley C, Kearney DL, Cooper TA (2005) Cardiac tissue-specific repression of CELF activity disrupts alternative splicing and causes cardiomyopathy. Mol Cell Biol 25: 6267–6278. 15988035 10.1128/MCB.25.14.6267-6278.2005 PMC1168813

[39] Berger DS, Moyer M, Kliment GM, van Lunteren E, Ladd AN (2012) Expression of a dominant negative CELF protein in vivo leads to altered muscle organization, fiber size, and subtype. PLoS ONE 6: e19274 doi:10.1371/journal.pone.0019274. 21541285 10.1371/journal.pone.0019274 PMC3082560

[40] Timchenko NA, Iakova P, Cai ZJ, Smith JR, Timchenko LT (2001) Molecular basis for impaired muscle differentiation in myotonic dystrophy. Mol Cell Biol 21: 6927–6938. 11564876 10.1128/MCB.21.20.6927-6938.2001 PMC99869

[41] Smibert CA, Wilson JE, Kerr K, Macdonald PM (1996) smaug protein represses translation of unlocalized nanos mRNA in the Drosophila embryo. Genes Dev 10: 2600–2609. 8895661 10.1101/gad.10.20.2600

[42] Jeske M, Moritz B, Anders A, Wahle E (2011) Smaug assembles an ATP-dependent stable complex repressing nanos mRNA translation at multiple levels. EMBO J 30: 90–103. 21081899 10.1038/emboj.2010.283 PMC3020108

[43] Tadros W, Goldman AL, Babak T, Menzies F, Vardy L, et al (2007) SMAUG is a major regulator of maternal mRNA destabilization in Drosophila and its translation is activated by the PAN GU kinase. Dev Cell 12: 143–155. 17199047 10.1016/j.devcel.2006.10.005

[44] Rouget C, Papin C, Boureux A, Meunier AC, Franco B, et al (2010) Maternal mRNA deadenylation and decay by the piRNA pathway in the early Drosophila embryo. Nature 467: 1128–1132. 20953170 10.1038/nature09465 PMC4505748

[45] Andrews S, Snowflack DR, Clark IE, Gavis ER (2011) Multiple mechanisms collaborate to repress nanos translation in the Drosophila ovary and embryo. RNA 17: 967–977. 21460235 10.1261/rna.2478611 PMC3078745

[46] Baez MV, Luchelli L, Maschi D, Habif M, Pascual M, et al (2011) Smaug1 mRNA-silencing foci respond to NMDA and modulate synapse formation. J Cell Biol 195: 1141–1157. 22201125 10.1083/jcb.201108159 PMC3246892

[47] Furling D, Doucet G, Langlois MA, Timchenko L, Belanger E, et al (2003) Viral vector producing antisense RNA restores myotonic dystrophy myoblast functions. Gene Ther 10: 795–802. 12704419 10.1038/sj.gt.3301955

[48] Langlois MA, Lee NS, Rossi JJ, Puymirat J (2003) Hammerhead ribozyme-mediated destruction of nuclear foci in myotonic dystrophy myoblasts. Mol Ther 7: 670–680. 12718910 10.1016/s1525-0016(03)00068-6

[49] Mulders SA, van den Broek WJ, Wheeler TM, Croes HJ, van Kuik-Romeijn P, et al (2009) Triplet-repeat oligonucleotide-mediated reversal of RNA toxicity in myotonic dystrophy. Proc Natl Acad Sci U S A 106: 13915–13920. 19667189 10.1073/pnas.0905780106 PMC2728995

[50] Wheeler TM, Sobczak K, Lueck JD, Osborne RJ, Lin X, et al (2009) Reversal of RNA dominance by displacement of protein sequestered on triplet repeat RNA. Science 325: 336–339. 19608921 10.1126/science.1173110 PMC4109973

[51] Lee JE, Bennett CF, Cooper TA (2012) RNase H-mediated degradation of toxic RNA in myotonic dystrophy type 1. Proc Natl Acad Sci U S A 109: 4221–4226. 22371589 10.1073/pnas.1117019109 PMC3306674

[52] Francois V, Klein AF, Beley C, Jollet A, Lemercier C, et al (2011) Selective silencing of mutated mRNAs in DM1 by using modified hU7-snRNAs. Nat Struct Mol Biol 18: 85–87. 21186365 10.1038/nsmb.1958

[53] Foff EP, Mahadevan MS (2011) Therapeutics development in myotonic dystrophy type 1. Muscle Nerve 44: 160–169. 21607985 10.1002/mus.22090 PMC3136655

[54] Fernandez-Funez P, Nino-Rosales ML, de Gouyon B, She WC, Luchak JM, et al (2000) Identification of genes that modify ataxin-1-induced neurodegeneration. Nature 408: 101–106. 11081516 10.1038/35040584

