# Supplementary figures and images for: Smaug/SAMD4A Restores Translational Activity of CUGBP1 and Suppresses CUG-Induced Myopathy
Source: PLoS Genet. 2013 Apr 18;9(4):e1003445. doi: 10.1371/journal.pgen.1003445 (PMC3630084; doi:10.1371/journal.pgen.1003445)

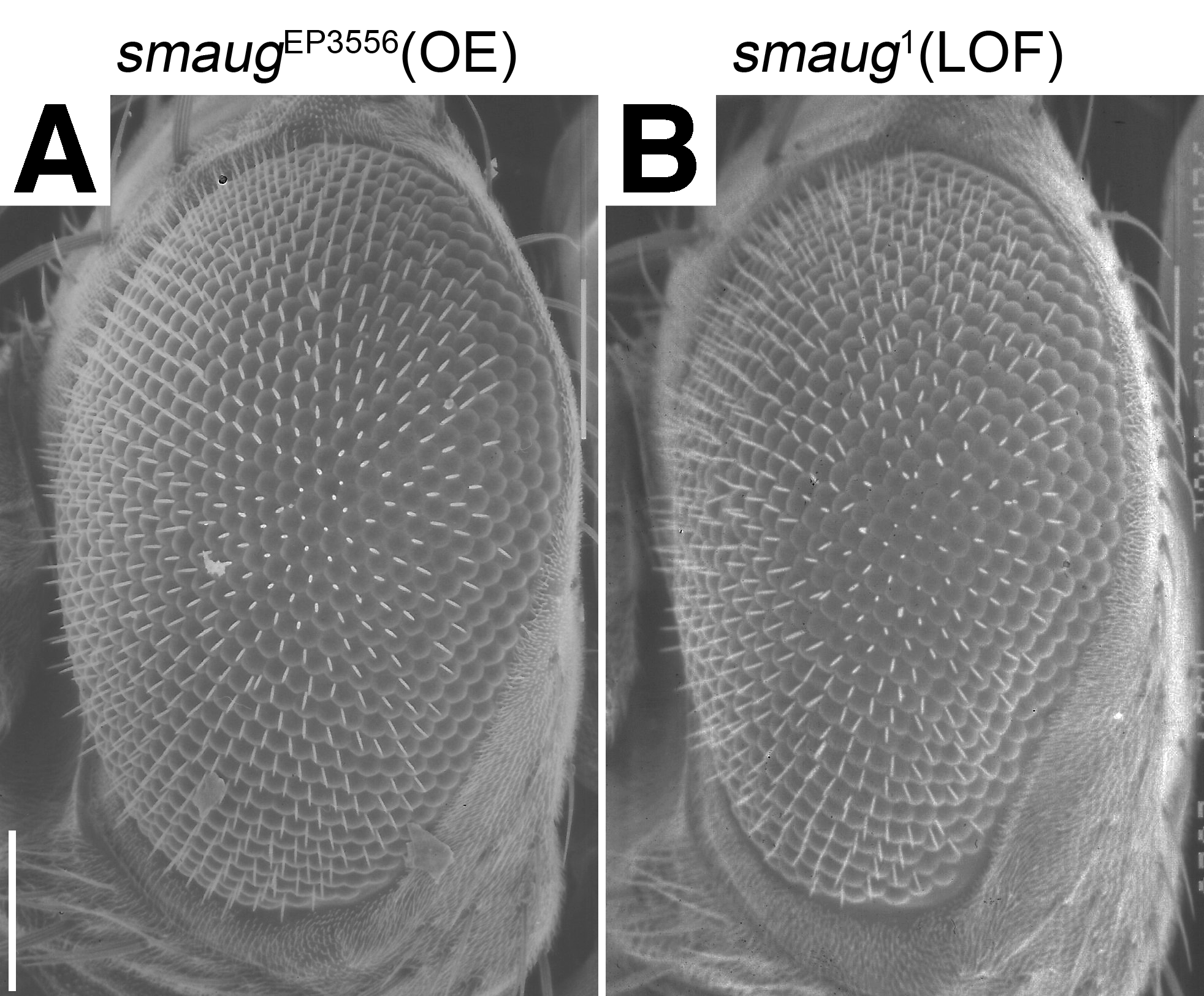

Supplement: Figure S1 — Overexpression and loss-of-function alleles of smaug do not modify the phenotype of control eyes. A–B. Neither overexpression (A), nor loss-of-function (B) alleles of smaug in heterozygosity cause an abnormal phenotype in control eyes (GMR-Gal4). Genotypes: A: w; GMR-Gal4/+; smgEP3556/+. B: w; GMR-Gal4/+; smg1/+. Scale bar: 100 µm. (TIF) [file pgen.1003445.s001.tif]

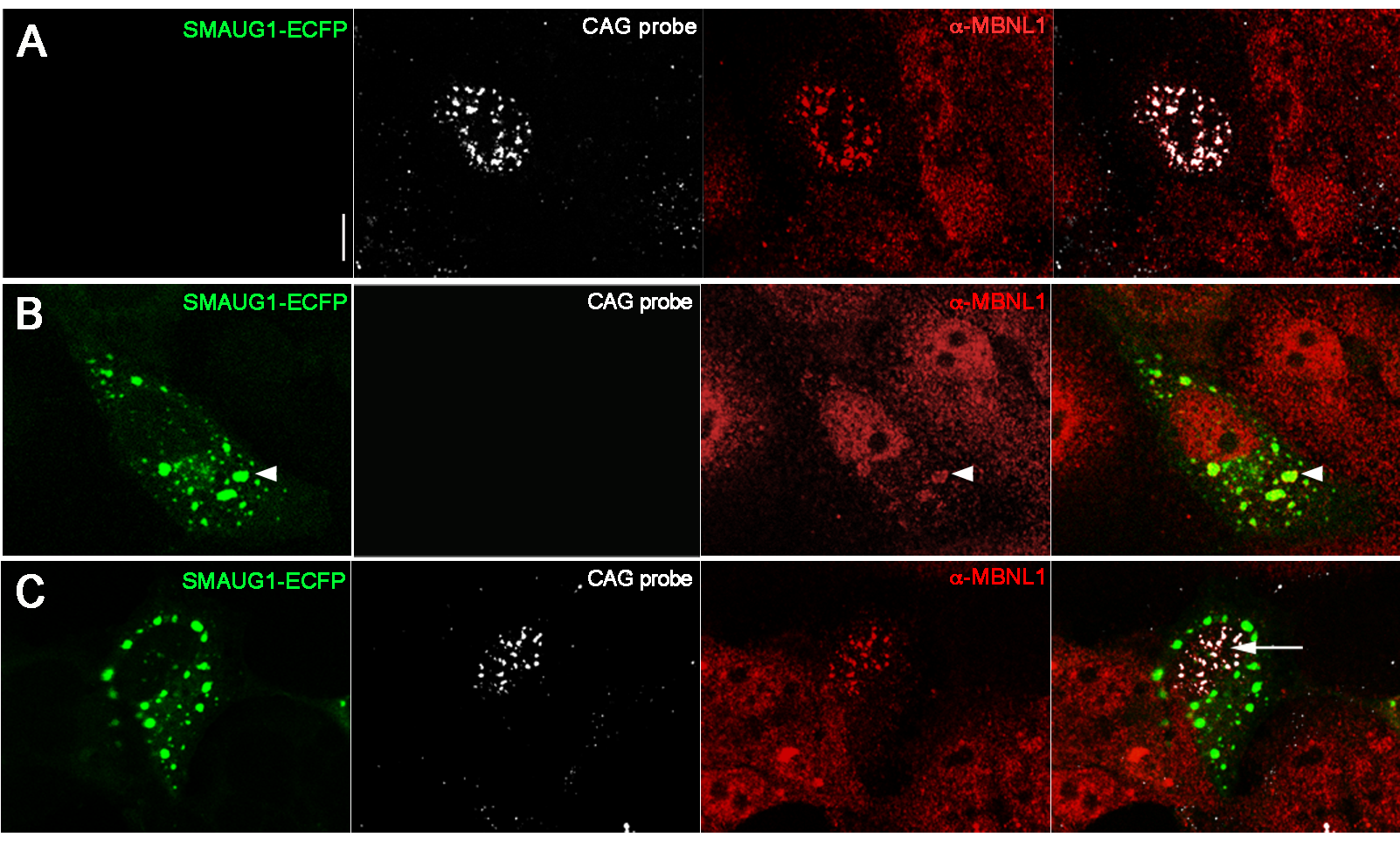

Supplement: Figure S2 — MBNL1 sequestration in nuclear foci is not altered by SMAUG1 overexpression. Immunofluorescense and in situ images of COSM6 cells A. In cells transfected with (CUG)960, the expanded CUGs accumulate in the nuclear foci (CAG probe, white), where they sequester endogenous MBNL1 (α-MBNL1, red). B. In cells transfected with SMAUG1-ECFP, SMAUG1-ECFP (green) accumulates in the cytoplasm, where it co-localizes with MBNL1 (arrowheads) (α-MBNL1, red). C. In cells cells co-transfected with (CUG)960 and SMAUG1-ECFP, sequestration of MBNL1 (α-MBNL1, red) by CUG nuclear foci (CAG probe, white) is shown by co-localization of both (arrow). Note that co-localization of MBNL1 and CUG nuclear foci is not altered by SMAUG1-ECFP (green) expression (compare panels A and C). Scale bar: 10 µm. (TIF) [file pgen.1003445.s002.tif]

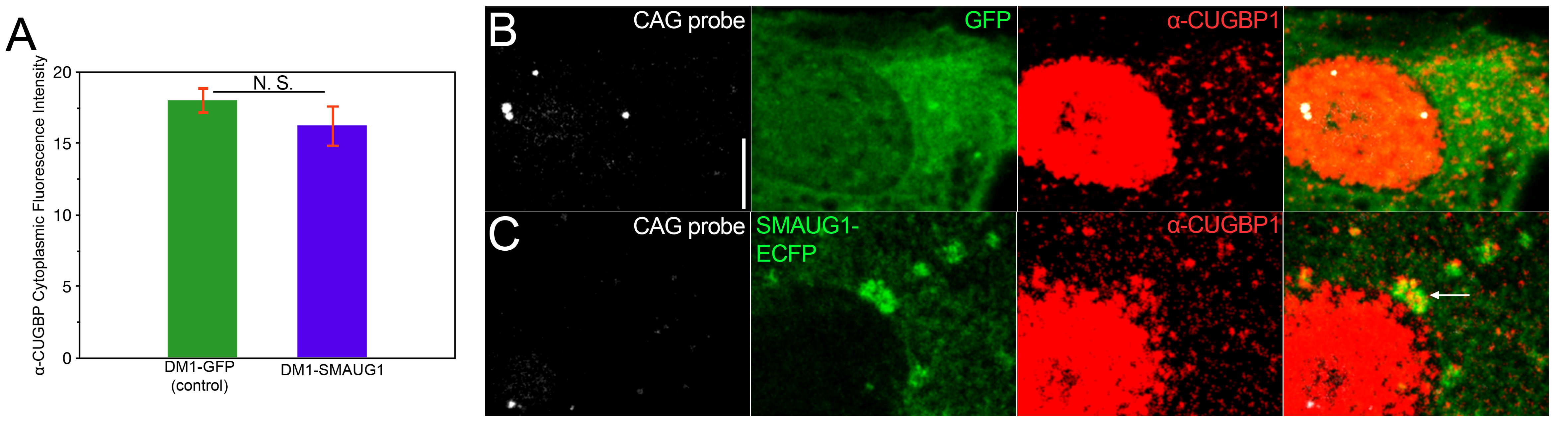

Supplement: Figure S3 — Expression of SMAUG1 in DM1 human myoblasts does not affect cytoplasmic CUGBP1 levels. A. Bar graph representing the intensity of α-CUGBP1 cytoplasmic signal in DM1 myoblasts transfected with GFP (DM1-GFP, green bar), versus DM1 myoblasts transfected with SMAUG1 (DM1-SMAUG1, blue bar). Data was analyzed with ANOVA followed by Student's t test; NS: not significant. B–C. Representative images of DM1 myoblasts transfected with GFP (green) (B) or SMAUG1 (SMAUG1-ECFP, green) (C) taken at high exposure to reveal cytoplasmic CUGBP1. Note that the intensity of cytoplasmic CUGBP1 (α-CUGBP1, red) is similar in both cases. See also co-localization of CUGBP1 and SMAUG1 (C, arrow). Scale bar: 10 µm. (TIF) [file pgen.1003445.s003.tif]

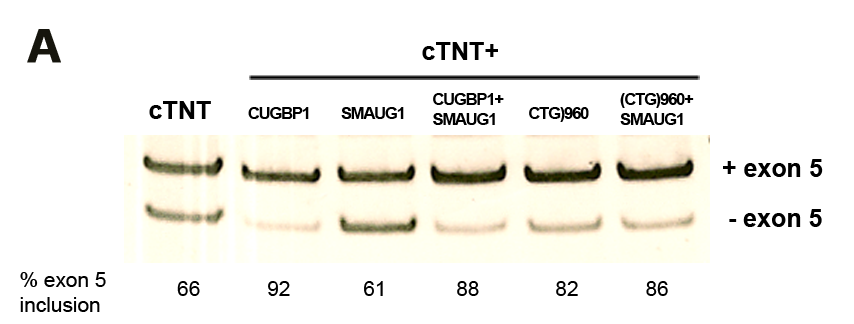

Supplement: Figure S4 — SMAUG1 does not modify splicing of cTNT minigene. A. SMAUG1 does not modify splicing changes caused by CUGBP1 or (CTG)960 on cTNT minigene. CosM6 cells transfected with cTNT minigene show 66% exon 5 inclusion. Upon co-transfection with CUGBP1 or (CTG) 960 exon 5 inclusion increases to 92 and 82% respectively. This splicing pattern is not affected by SMAUG1 transfection. Expression of SMAUG1 alone with the minigene has a similar splicing pattern to cTNT minigene alone with 61% exon inclusion. (TIF) [file pgen.1003445.s004.tif]

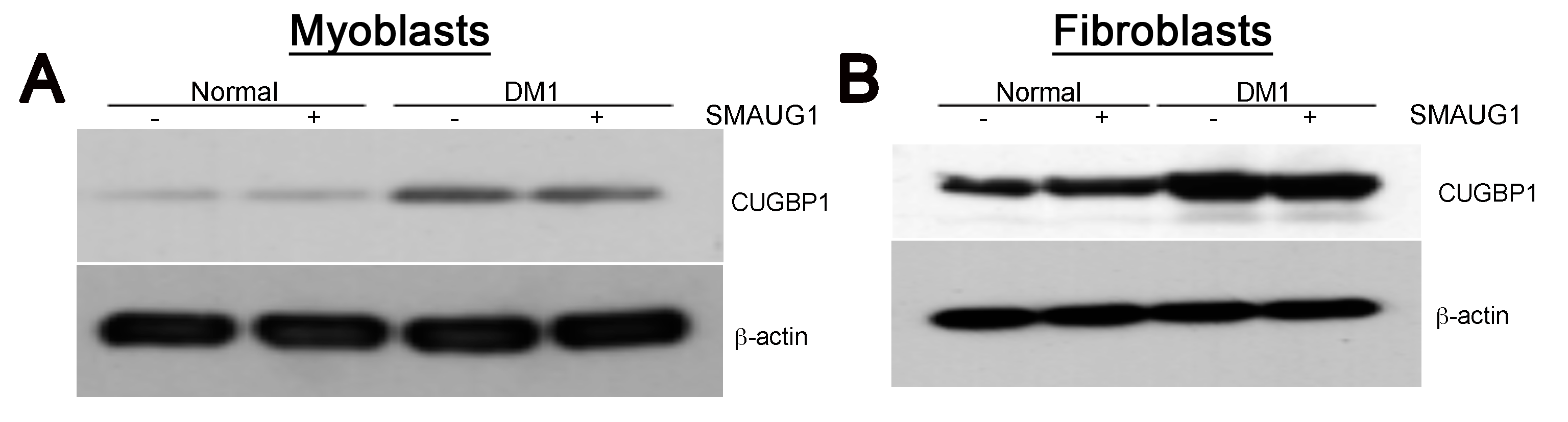

Supplement: Figure S5 — SMAUG1 does not affect abnormally elevated levels of CUGBP1 in the cytoplasm. A. Western blot analysis of protein levels from cytoplasmic extracts in control and DM1 myoblasts. CUGBP1 levels are increased in DM1 myoblasts. B. Western blot analysis of protein levels from cytoplasmic extracts in control and DM1 fibroblasts. CUGBP1 levels are increased in DM1 fibroblasts. β-Actin used as control. (TIF) [file pgen.1003445.s005.tif]

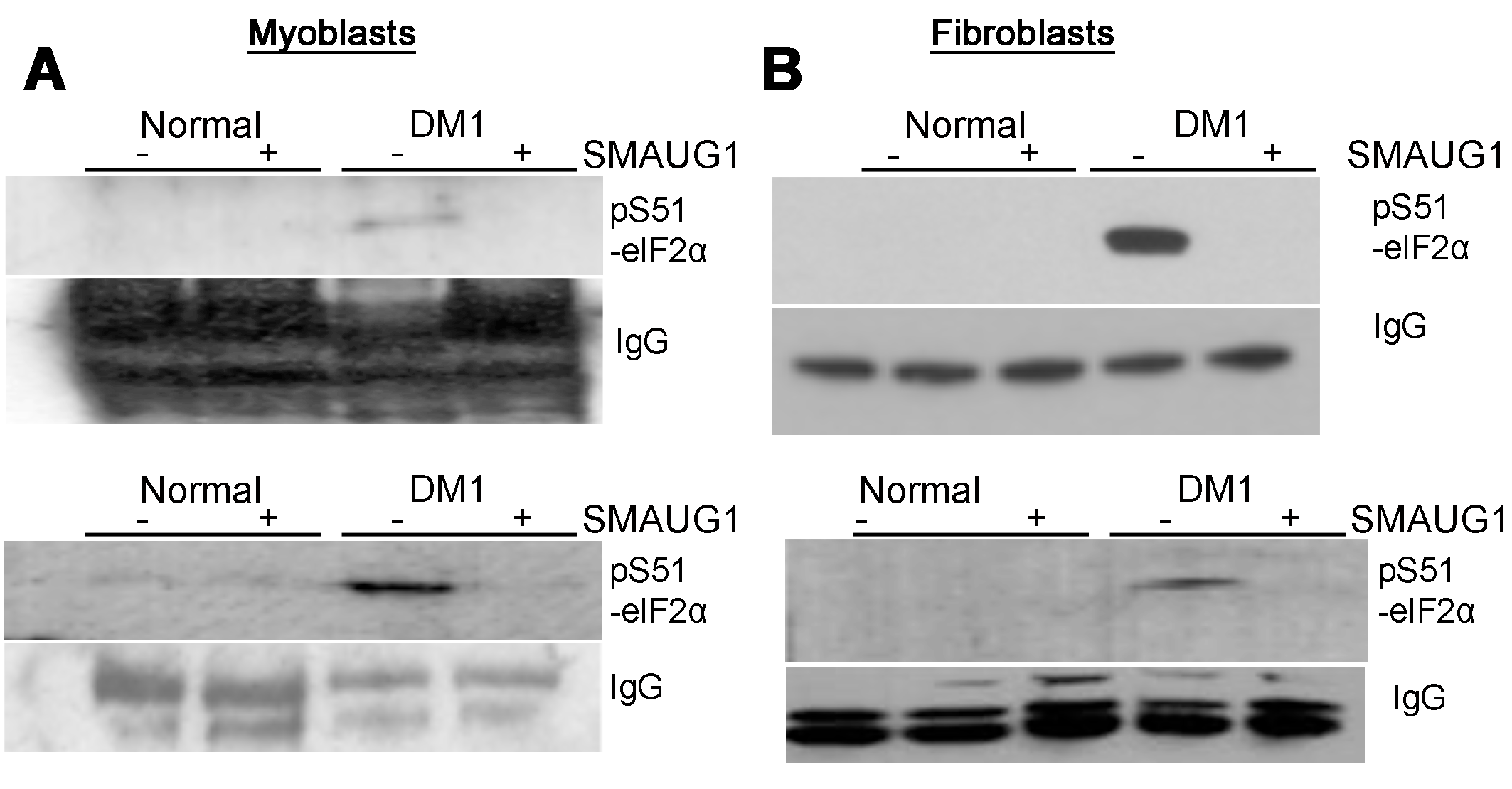

Supplement: Figure S6 — Reproduction of the observation that SMAUG1 reduces inactive CUGBP1/pS51-eIF2α translational complexes. A. Cytoplasmic protein extracts from normal and DM1 myoblasts immunoprecipitated with CUGBP1 antibody was probed with antibody to specific inactive pS51-eIF2α (CUGBP1 IP) in two additional experiments with similar results. B. Cytoplasmic protein extracts from normal and DM1 fibroblasts immunoprecipitated with CUGBP1 antibody was probed with antibody to specific inactive pS51-eIF2α (CUGBP1 IP) in two additional experiments with similar results. Note that inactive pS51-eIF2α is undetectable after SMAUG1 transfection. IgG, heavy chains of immunoglobulins detected on the same filter. (TIF) [file pgen.1003445.s006.tif]

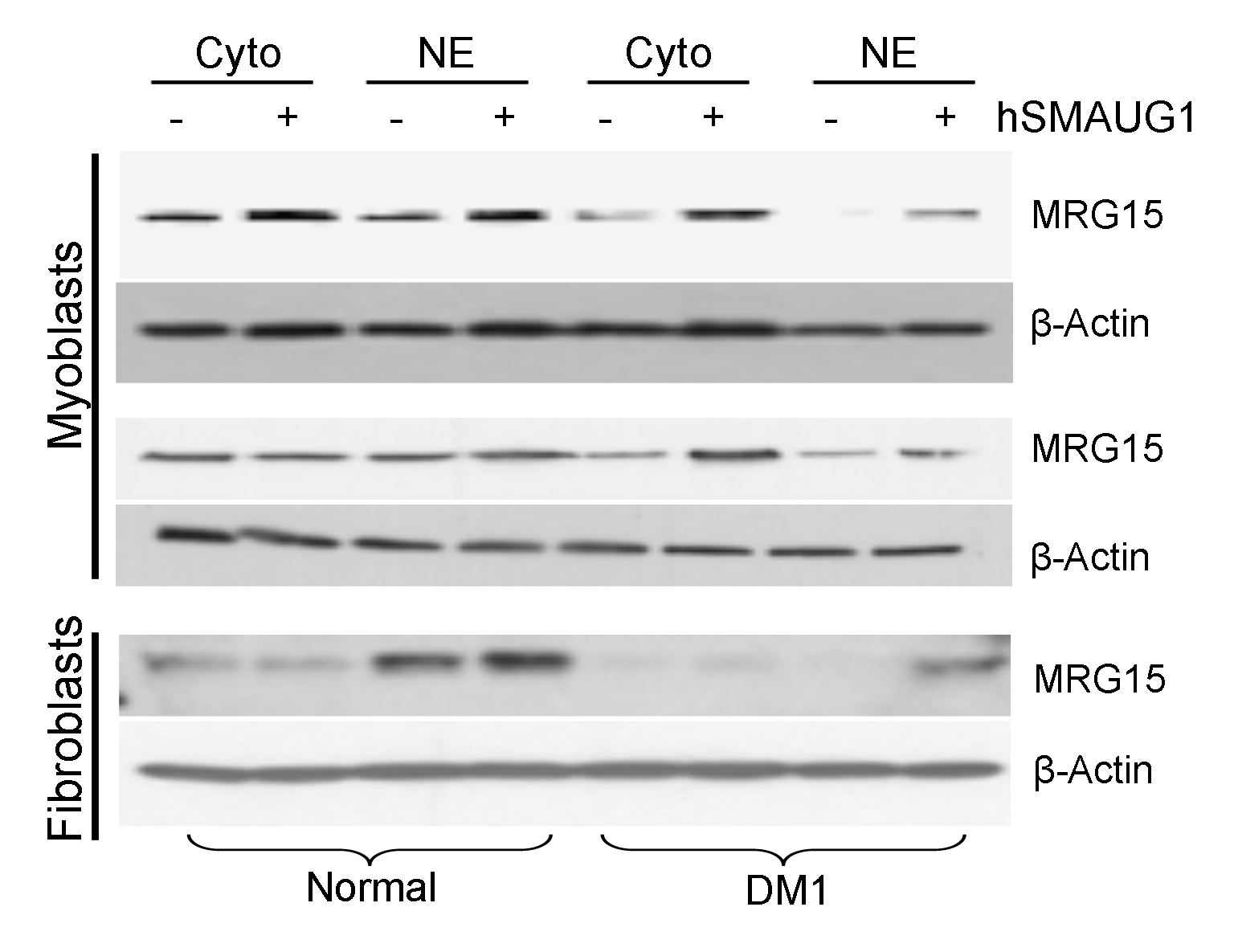

Supplement: Figure S7 — Reproduction of the observation that SMAUG1 recuperates normal levels of MRG15 protein in DM1 myoblasts and fibroblasts. SMAUG1 recuperates normal levels of MRG15 protein in DM1 myoblasts and fibroblasts. Nuclear and cytoplasmic proteins of normal and DM1 myoblasts and fibroblasts were examined by Western blotting with antibodies to MRG15. The filter was re-probed with antibodies to β-actin. (TIF) [file pgen.1003445.s007.tif]
